# Enabling manufacturing firms' supply chain performance in the Middle East region through boosting the quality of multi-directional relationship, and supply chain risk dilution: A moderated-mediation model^☆^

**DOI:** 10.1016/j.heliyon.2023.e22059

**Published:** 2023-11-09

**Authors:** Moh'd Anwer AL-Shboul

**Affiliations:** Business Administration Department, King Talal School of Business Technology, Princess Sumaya University for Technology (PSUT), P.O. Box 1438 Al-Jubaiha, Amman, Jordan

**Keywords:** Elasticity, Dependability, Multi-directional quality relationship, Strategic supplier partnership, supply risk dilution, manufacturing firms, SC performance, Middle East region

## Abstract

Elasticity and dependability factors are considered fundamental elements that have a significant and direct impact on reducing waste in manufacturing companies' resources as well as their supply chains (SCs) in order to improve their overall performance, as well as improving the performance of these companies' supply chains. This is in addition to not overlooking the effective and important strategic relationship and the role of suppliers to manufacturing companies as a mediating factor in the relationship between the qualities of the multi-directional relationship and mitigating the risks that it may be exposed to during its operational and production process. 156 samples were included in this study out of 743 small to large manufacturing companies in the Middle East region, including Jordan, Turkey, Saudi Arabia, and Egypt, where an experimental examination was conducted of the companies targeted in this study to prove or reject the proposed hypotheses. Structural equation modeling was applied and used to examine the proposed hypotheses. The results of this study indicated that the elements of flexibility and dependability have positive, direct, and statistically significant effects on mitigating supply risks, and this therefore works to improve the performance of the supply chain of manufacturing companies, especially small and medium-sized companies. In addition, the results of this study showed that building strong strategic partnerships with dependable, certified, high-performance, and flexible suppliers has a positive impact on moderating the potential supply risks that manufacturing companies may face in their operational processes, while the results showed that there is no statistically significant effect on the relationship between dependability and mitigate supply risks. This research paper offers empirical evidence for using the quality of the multi-directional relationship within supply risk dilution of manufacturing firms’ context in developing countries for enhancing their supply chain performance. This study provides a clear roadmap and basis for managers and decision-makers in manufacturing companies to focus on the importance of the quality of relationships with suppliers and business partners in their business dealings to limit and mitigate the risks that they may face, which may lead to interruption of the necessary supplies to them.

## 1 Introduction

Customer retention is vital for businesses worldwide due to the heightened competition in the ever-changing marketplace [1,2]. As a result, numerous manufacturing firms have implemented key measures to improve their performance and that of their supply chain partners, aiming to foster strong, close relationships with their trading counterparts to mitigate potential uncertainties during business operations. During the past period, trends towards producing unique quality products have accelerated outsourcing multiple activities to their suppliers [3].

Within the supply chain management (SCM) literature, the quality of the multi-directional association (QoMDR) between buyers and suppliers has been the focus of numerous research studies. Several previous studies focused on the importance of the link between the flexibility of manufacturing companies and the speed of responding (i.e. agility) to signals that come from different markets, and the ability to respond promptly to customer demands and ensure timely delivery [4]. Furthermore, effective communication among manufacturing firms can result from well-established relationships between these organizations [5,6]. Some research has also assessed the effect of QoMDR on SCP [e.g., 32, 98]. Overall, the buyer-supplier QoMDR can be viewed as the mutual satisfaction both parties derive from their interactions. In this context, maintaining an appropriate level of QoMDR between buyers and suppliers can help sustain their relationship, subsequently enhancing efficiency, effectiveness, and productivity [7,8,9,10,11,and99]].

The literature highlights various dimensions for evaluating the quality of buyer-supplier multi-directional relationships. Elasticity and dependability are often regarded as crucial elements in such assessments [12,13,and^14^]]. As a result, this factor was examined and tested in relation to other dimensions included in the study model. Therefore, strong relationships can be built between approved suppliers and buyers from manufacturing companies that improve their efficiency by applying several important factors in the relationship, such as speed of response, dependability, flexibility, and trust [15]. This is in addition to the fact that increasing differences in cultural factors may play a negative role in expanding partnerships and increasing delays in delivering supplies, which leads to a steady increase in costs through searching for new suppliers. This prompted most manufacturing companies to search for approved suppliers by building the quality of the relationship, which is considered a crucial factor in simplifying the operational processes of manufacturing companies [16,17,and79]].

Moreover, elasticity and dependability in buyer-supplier associations play a key role in mitigating opportunistic behavior within manufacturing firms [18,10,and104]]. Furthermore, strong connections relying on the dependability of the SC partners might significantly reduce the buyer's perception of supply disruption risk [19,20,and^21^]]. The purpose of this research is to investigate the effects of multi-directional association quality dimensions on a manufacturing firm's SCP, particularly through the lens of supply risk reduction [22,23].

The concept of supplier strategic partnership (SSP) encompasses the degree to which suppliers offer raw materials, components, items, and information, to assist the manufacturing firms in order to enhance the level of cooperation with them in their operational processes, either partially or entirely, to address current and future customer needs [24,25]. In the realm of SCM literature, various studies have explored different aspects of SSP, including its role in elasticity and dependability [26,27,and67]]. Its benefits and drawbacks [28,16], the appropriate timing for SSP [29,30], and supplier interfaces in the context of technological advancements, elasticity, and dependability [31,32].

When resorting to implementing an emergency plan, it is necessary to review the list of the company's main resources mainly relying on resource-based review theory. The empirical study conducted by Ref. [33] emphasized the importance of the smooth and sustainable flow of resources for manufacturing companies by relying on several reliable and accredited sources. This certainly increases the versatility and flexibility of companies and their ability to compete and produce by alleviating disruptions that may occur through their supply chains. Study [34] focused and pointed to the effective role played by supply chains, especially the green approach for manufacturing companies, in order to increase their competitive capabilities. Thus, formulating, maintaining, and adopting a long-term association between buyers and dependable suppliers has drawn growing focus from many researchers on the topics related to the volume of trade exchange [e.g. 4, 48]. Many benefits can be achieved through high-quality multi-relationships with suppliers consisting of fostered elasticity and dependability [35], achieved value-added and appropriation [18,36,and^37^]], continuous successful works in firms [28], and leading super levels of the firm's SCP [5]. The quality of multi-directional relationships (i.e. elasticity, and dependability) between the focal firms and their emphasis has been placed on the fact that the true and effective strategic company between trading partners, including manufacturing companies and dependable suppliers, has a large and vital role in the volume of trade exchange between them with a long-term orientation. Therefore, this factor is considered a strategic weapon for many companies in obtaining a competitive advantage in addition to creating value for their operational operations and various logistical facilities in a diverse and rapidly changing environment [38]. In line with the increasing interest in boosting, the quality of multi-directional relationships with reliable and dependable suppliers, still, researchers paying major attention to unethical and opportunistic behavior that may appear from partners in supply chains. This behavior is considered inappropriate, illegal, and unethical, especially in trade exchange and cooperation operations between commercial parties, which include relationships between buyers and suppliers.

In order to overcome the factor of causal ambiguity that may occur from some trading partners in order to obtain some special benefits, including the companies' supply chains, the ideal procedure is to build relationships with partners within the supply chain network to build mutual trust, and of high quality, multi-directional and it may be an effective means of increasing aspects of cooperation and mutual exchange between all parties [39]. The association quality between buyers and suppliers plays a crucial and key role in enhancing a firm's performance and SCP as well [1]. Therefore, supplier relationship management with specific and significant features was considered an independent variable in many studies [40,41]. Thus, supplier relationships such as elasticity and dependability affect buyers' behaviors [4,42]. According to Ref. [11], a study found that positive supplier relations enhance competitive advantage by improving flexibility, and responsiveness [43]. specified the main features that contribute to supplier relationship practices such as flexibility and commitment. Thus, the quality of the relationship with suppliers can improve SCP excellence [25,40]. However, little attention was paid to the effect of the firms' quality of multi-directional relationships with its several suppliers. In order to fill the knowledge gap in the SCM literature, this study was applied to examine and test the association between firms' multi-directional quality relationships locally and even globally with their several suppliers with features such as elasticity, and dependability. Moreover, we include supplier strategic partnership in the study model as a moderator factor to examine the effect role in the linkages between the association of quality and supply chain risk dilution. Furthermore, this study also tries To fill the gap in studying the aforementioned factors that are related to the SCM literature and their applications, especially in the countries of the Middle East region in particular, which appear in the following points below.

➢ Referring to the SCM literature, the key factors obtained in this paper were not covered by the previous research, such as relying on the impact of multi-directional quality relationships on manufacturing firms' SCP directly and indirectly. Indirectly through the existing effect of the mediating factor (i.e., supply chain risk dilution), and through the moderating impact of supplier strategic partnership in the association between multi-directional quality relationships and supply chain risk dilution, and putting them in one study package and their impacts on SC risk dilution in the countries located in the Middle East region particularly.
➢ Most previous studies in the relevant literature have been applied to developed countries, while there are a few number that have been conducted on countries in the Middle East region. The dimensions addressed in this study and not addressed in previous studies are summarized in Table 1.

**Table 1:** Summarizes the items/factors that are included in this study and not included in other studies in the literature (Source: developed by Author).

Author(s)	Research area items/factors existing in this study and cross with other studies (yes/no)	Research area items/factors found in previous studies/the literature	Research Methodology	Findings	Study's contribution to the literature and not included in previous studies
Chiang and Wu (2016)	-Quality of multi-directional relationship/Yes -Supply risk dilution/No - Manufacturing firms' supply chain performance (MFSCP)/Yes -Supplier strategic partnership/No.	Relationship characteristics, relationship quality, and supply chain performance (SCP).	Mail survey, sample of 821 manufacturing companies in the electronics sector in the Republic of Ireland	Providing empirical support for effects of both duration and supplier awards being supported but degree of product standardization/customization and SC tier position not supported.	This study is conducted based on the recommendation of Fynes et al. (2008) for further future research to focus on the impact of other moderator variables (e.g., SSP on the SC relationship quality–MFSCP.
Bao et al. (2017)	-Quality of multi-directional relationship/Yes -Supply risk dilution/No -Manufacturing firms' supply chain performance (MFSCP)/Yes -Supplier strategic partnership/No	Buyer–supplier partnership quality, SCP, moderating role of demand and supply-side risks, and environmental uncertainty.	Web-based electronic survey used from 127 US firms.	The results demonstrate a positive relationship between partnership quality and SCP. Furthermore, evidence of higher demand side risk resulted in a stronger positive relationship between partnership quality and SCP.	This study included SRM as a mediating factor, not a moderator, and then show its impact on SCP, which added knowledge value to the literature.
El Baz and Ruel (2021)	-Quality of multi-directional relationship/Yes -Supply risk dilution/No -Manufacturing firms' supply chain performance (MFSCP)/Yes -Supplier strategic partnership/No	Relationship quality, SC operational performance and satisfaction with strategic performance.	Survey-based approach.	Relationship quality provides a global measure of buyer–supplier relationships and can be used to assess the types of relationships a firm has within its supply chain.	This study examined other factors such as SRM as mediating and SI as moderator and then show their impacts on SCP.
Soares et al. (2017)	-Quality of multi-directional relationship/Yes -Supply risk dilution/No -Manufacturing firms' supply chain performance (MFSCP)/Yes -Supplier strategic partnership/No	Supply chain management performance (SCMP), supply chain performance measurement (SCPM)	Data were compiled and collected from 213 operations and supply chain (SC) heads from leading retail stores in India.	Statistical tests show that the implementation of SCMP are associated with supply chain performance measures, which leads to overall improvements; moreover, there is a statistically significant association between the five SCMP and eight SCPM.	This study investigated SRM as a mediating factor and SI as moderator, which are not include in Gawankar et al. (2017) study.
Yan et al. (2018)	-Quality of multi-directional relationship/Yes -Supply risk dilution/No -Manufacturing Firms' supply chain performance (MFSCP)/No -Supplier strategic partnership/No	Relationship quality (top management support and relational governance), supplier development, green supply chain integration, cost driver, customer driver.	285 samples collected from ten countries.	The results show that supplier development fully mediates the relationship between top management support and upstream GSCI and partially mediates the relationship between relational governance and upstream GSCI. Additionally, both cost and customer drivers are found to significantly moderate the relationship between supplier development and upstream GSCI.	This study focused on QoR (i.e., confidence and reliance) not as factors included in Lo et al. (2018) study (i.e., top management support and relational governance), further focused on SI as a moderator factor in comparison with supplier development as a mediating factor and its impact on upstream green SC integration in Lo et al. (2018) study.
Wang et al. (2021a)	-Quality of multi-directional relationship/Yes -Supply risk dilution/No -Manufacturing Firms' supply chain performance (MFSCP)/Yes -Supplier strategic partnership/No	Supply chain relationship quality (SCRQ), firm performance (FP), supply chain management performance (SCMP), supply chain performance (SCP)	Survey data from manufacturing companies	Effective management of SCRQ, SCMP and SCP can provide better FP and a competitive advantage,	This study assessed SRM as a mediating factor, and SI as a moderator factor, then show their impacts on SCP, which are not included in the study of Yumurtaci et al. (2020); thus, it added knowledge value to the literature.
Wang et al. (2021b)	-Quality of multi-directional relationship/Yes -Supply risk dilution/No -Manufacturing Firms' supply chain performance (MFSCP)/Yes -Supplier strategic partnership/No	Intrafirm relationship quality (relational, operational, information), SCP (financial and market share, operational, and relational).	Total of 210 papers were reviewed in the literature, and a total of 100 papers remain for further analysis.	Relational dimension plays a key role in SC relationship management and influences performance significantly. Information dimension will affect performance indirectly through relational dimension	This study examined SRM as a mediating factor, and SI as a moderator factor, then show their impacts on SCP, which are not included in the study of Qian et al. (2021); thus, it added knowledge value to the literature.
Aghazadeh et al. (2022)	-Quality of multi-directional relationship/Yes -Supply risk dilution/No -Manufacturing Firms' supply chain performance (MFSCP)/No -Supplier strategic partnership/No	Collaborative buyer-supplier relationships, social sustainability, moderating effects of justice and big data analytical intelligence.	Using primary survey data collected from supply chain practitioners in South Africa.	Collaborative buyer-supplier relationships positively influence supplier social sustainability in the new normal era. However, it is relatively stronger from the suppliers' perspective when compared with the buyers' perspective. Secondly, the moderating effect of perceptions of organizational justice and big data analytical intelligence on the relationship between collaborative buyer-supplier relationships and supplier social sustainability is also statistically significant. However, it is relatively stronger from the buyers' perspective when compared with the suppliers' perspective.	This study focused on SRM as a mediating factor, SI as a moderator factor, and their impacts on SCP in comparison with justice and big data analytical intelligence assimilation as moderating factors and their impacts on social sustainability in the new-normal era in the study of Bag et al. (2022).
Wang et al. (2023)	-Quality of multi-directional relationship/Yes -Supply risk dilution/No -Manufacturing Firms' Supply chain performance (MFSCP)/No -Supplier strategic partnership/No	Relatonship quality, collaborative (c) commerce behavior, firms' dynamic capability, and c-commerce performance.	Survey data collected from 257 professionals in various manufacturing industries.	Collaborative -commerce behavior and dynamic capability are positively associated with c-commerce performance. However, negative calculative commitment does not positively affect c-commerce behavior. Moreover, institution-based trust does not negatively affect negative calculative commitment.	This study shared only in QoR factor with Wang et al. (2023) study. While all other tested, factors in this study are not included in the study of Wang et al. (2023). Thus, this study has added other sights and knowledge to the literature.

This study is structured as follows: the literature background the study model and the proposed hypotheses, which are presented in Section 2. Section 3 illustrates the approach and clarifies the findings of statistical analysis. Section 4 explains the data analysis and findings. Finally, the conclusion, managerial and theoretical implications, main contributions, limitations, and future research work are obtained in Section 5.

## 2 Literature background and conceptual research model hypotheses

There are many definitions that have been discussed in multi-directional relationships, especially those included in the relationship between buyer and supplier, where emphasis has been placed on the importance of QoMDR in building the relationship, which leads to levels of satisfaction for each party over the other. The study of both [44,45] confirmed that there are several important elements and characteristics that must be included in the relationship in order for the relationship to be strong and clear, such as flexibility, elasticity, trust, and dependability. They believe that optimal levels of these factors can lead to richness, effectiveness, and performance, ultimately fostering collaboration and achievement [46,43]. mentioned in developing and building business-to-business associations that customer satisfaction, reliability, elasticity, and dependability as fundamental elements of QoMDR [47]. emphasize relationship quality, considering it the most appropriate element for meeting the needs of customers involved in the relationship [13]. suggest the quality might include a multi-dimensional component, which obtained many features to assess it [48]. focused on building and strengthening high-quality communications and relationships with customers so that they turn into highly loyal customers as a goal in building relationships based on an important element, which is QoMDR [49]. focused of their study on the quality of relationships, laying the groundwork for this concept. Since 1995, numerous scholars have investigated the quality of relationships [50], examining different factors to examine association quality [22,51,and88]]. Researchers such as [52,46,53], and [54] have used trust and dependability as the quality of association measurement dimensions. Several studies, including those by Refs. [55,46,56,57,9,58], and [11], have examined trust, commitment, dependability, and satisfaction as the quality of relationship factors. Further elements such as timeliness, customer familiarity, ethical boundaries, understanding of collaborative patterns among resellers [4], influential opposition [55], connection disputes [59], adaptation, interaction, coordination, and perception [60], and service quality [46] have been studied. Other research has investigated variables like clashes, coordination, restrictions, and social barriers to assess the quality of the relationship. A broader range of quality dimensions has been proposed by a number of previous studies, which dealt with this dimension from several different points of view, such as service quality, logistical transport quality, communication quality, cooperation quality, and interaction quality [42]. In this study, the operational definition of the quality of multidimensional relationship is composed of satisfaction, flexibility, elasticity, cooperation, collaboration, coordination, commitment, customer orientation, ethical profile, trust, the intensity of communication, long-term orientation, and social and economic satisfaction of a focal actor in a business relationship.

This study mainly adopts the relational view theory (RVT), which focuses on the involvement of the quality of buyer-supplier relationships from a B2B perspective as a competitive strategy for the enhancement of a firm's performance and capabilities. It includes different perspectives and there are many features such as trust, commitment, flexibility, agility, green, elasticity, responsiveness, dependable, collaborative, and confident [53,61,and^62^]]. This study adopts such a theory relying on some previous research studies, which demonstrates the competitive advantages that the product-manufacturing firms obtained from building and formulating sustainable quality, flexible, and dependable relationships with reliable external partners locally and even globally such as [18] study. According to Ref. [18], and (2017), product-manufacturing firms that take a relational view should decide to involve external suppliers; they should have some specific significant features and main configurations of involvement in the relationships such as commitment, trust, reliability, flexibility, dependability, etc. in order to reduce risks and costs, wasting resources, and enhancing their performance as well. Furthermore, they mentioned the three key ingredients of involvement such as black, gray, and white boxes. These different possible features in the relationships between product manufacturing firms and service suppliers have been little examined in the SCM quality relationships literature, particularly in developing countries. Thus, most empirical studies have focused on the external suppliers' involvement as a make-build-or-buy decision perspective, without taking into consideration differences in types of cooperation and their implications for servitization [e.g. 109, and 110]. Therefore, as mentioned by Ref. [18], and (2021), the form of quality service of external dependable supplier involvement might have a vital effect on product manufacturing firms' functions and processes, on supply chain risk dilution, and on enhancing manufacturing firms' performance obtained from their servitization. Thus, this study investigates certain features in building and formulating quality relationships with strategic supplier participation, which might be more significant than other factors for the servitization strategy of product-manufacturing firms, which is mentioned particularly by the elasticity and dependability of their external suppliers through their services.

Therefore, this paper tries to respond to this question “To how much extent does the quality of the multi-directional relationship, supply risk dilution, and strategic supplier partnership have a crucial effect on the manufacturing firms' SCP particularly in countries located in the Middle East region?” Thus, formulating, maintaining, and adopting a long-term association between buyers and dependable suppliers has drawn growing focus from many researchers on the topics of commercial exchange connections [e.g. 4, 48]. Many benefits can be achieved through high-quality multi-relationships with suppliers consisting of fostered elasticity and dependability [35], achieved value-added and appropriation [18], continuous successful works in firms [28], and leads to super levels of the firm's SCP [5]. The quality of multi-directional relationships (i.e. elasticity, and dependability) between the focal firms and their dependable suppliers' factor was considered an effective and important element for the success and improvement of the relationship between business partners, especially in long-term business deals. Therefore, this factor was considered crucial, necessary, and effective for manufacturing companies in order to achieve competitive and comparative advantages and create added value to their operational processes and various activities in a rapidly changing and unstable environment [38]. In line with the increasing interest in boosting the quality of multi-directional relationships with reliable and dependable suppliers, still, researchers paying major attention to supply chain parties might have opportunistic behavior, which has been highly emphasized by many previous studies as a key parameter in commercial exchanges between buyers and their dependable suppliers.

Therefore, in order to overcome the causal ambiguity that may occur by one of the parties to the trade exchange to obtain some personal benefits at the expense of the trading partners, adopting a high-quality, multi-directional relationship for manufacturing companies, relying on several approved supply sources, will be an effective means to increase and raise the level of behavior cooperation between parties to trade [12,39]. The relationship quality between buyers and suppliers plays a key role in enhancing a company's performance and SCP as well [1]. Therefore, supplier relationship management with specific and significant features was considered an independent variable in many studies [10,41]. Thus, supplier relationships such as elasticity and dependability affect buyers' behaviors [4,42]. According to Ref. [11], a study found that positive supplier relations enhance competitive advantage by improving flexibility, and responsiveness [54]. specified the main features that contribute to supplier relationship practices such as flexibility and commitment. Thus, the quality of the relationship with suppliers can improve SCP excellence [40].

Elasticity and flexibility have been widely utilized in various research fields to evaluate relationship quality. It can be seen as a firm's willingness to depend on its external dependable partners in an elastic manner [63,11]. This factor originates from relationship marketing research, which identifies an external party's values, behaviors, and attitudes. Three aspects can be attributed to elasticity: 1) recognizing that the business partner demonstrates goodwill in their actions, 2) faith and assurance for the firm when relying on the business collaborator and 3) believing that a business partner could act in the best interest of all parties involved [59]. [23] defines elasticity as the extent of support given to a business partner, which results in increased productivity, effectiveness, and risk reduction. Moreover [22,51], Elasticity has been described as the company's agility, flexibility, and belief in its ability to maneuver its operational operations through joint coordination with its business partners and their ability to meet their demands with great cooperation through reliable supply sources [19]. mentioned the element of trust was considered an important factor in the success of a service-based relationship, and they view it as a key factor in establishing a sense of security in supplier relationships and fostering customer loyalty. Additionally, most studies in this area regard elasticity and dependability as essential factors for measuring relationship quality [64,31,1,and104]].

The concept of dependence originates from relationship marketing literature, which seeks to create and maintain a long-term relationship based on elasticity, flexibility, and reliance on others [22], and [51] identified emotional dependence, commitment, and functional dependence as the three dimensions of reliance [59]. emphasized that dependence represents manufacturing firms' inclination to enhance and maintain enduring relationships. This positive impact on participants' relationships leads to reduced illegal benefits. From the point of buyer's view, the reliance and dependability in the buyer-supplier relationship suggests maintaining contact with the supplier, while being open to ending the association if another competitor might offer dependable services [74, 80). According to Ref. [46], reliance and dependency are a firm's inclination to remain in a connection with its suppliers. Dependence in a relationship implies maintaining ongoing connections with other supply chain stakeholders.

Furthermore, dependence represents the desire to safeguard relationships [65]. Dependence is vital for a successful relationship because, without elasticity and dependability, manufacturing firms (MFs) and their supply chain become susceptible [65,3]. Maintaining a relationship founded on elasticity and dependence can affect the performance of MFs within their SCs [66]. Supplier embroilment and strategic partnership is seen as a cross-disciplinary phenomenon, addressed and explained in numerous research publications across various fields of study. such as obtaining R&D, technology advancements [e.g., 31] supply chains for RMs, semi-finished goods, and items [e.g., 44]; industrial marketing management [e.g., 90]; improving genuine product [e.g., 76]; and operations and manufacturing management [e.g., 74]. Many researchers have emphasized that different areas have examined the interference phenomenon between buyer-supplier linkages, using various concepts and perspectives for example win-win relationships, in-depth partnerships, trust, and full cooperation [5]. While these concepts are often applied in an exchangeable manner, still the definitions are not accurately standardized. Supplier strategic partnership and participant is the only one that is mentioned in specific definition and has represented numerous research topics addressing the interface between customers and suppliers [66]. Therefore, this term indicates that customers have the power with whom to go ahead to collaborate and cooperate and to go with strategic partnerships under various circumstances [67,68].

In the realm of supply risk, various definitions have been proposed [23]. featured supply risks such as supply shortages, dilution, techniques advancements, stand-by components, entry restrictions, logistical costs, and complex situations. Diverse approaches pertain to any circumstances that hinder the introduction of a new product or disrupt production [65]. [42] mentioned that risks are clustered into two classes. The first class is demand risk, which encompasses all actual risks to the supply of manufacturing firms' resources by suppliers, while the second is supply risk, which includes interruptions of supply [19]. classified SC risks into several forms due to different causes, one of these is supplier risk, which might happen by the upstream partners and refers to interruptions of procurement supply sources, providers, and distribution channels [69]. Moreover [[70], [71]], described supply risk as the risk a buyer faces when a supplier fails to fulfill supply commitments.

**Fig. 1:**
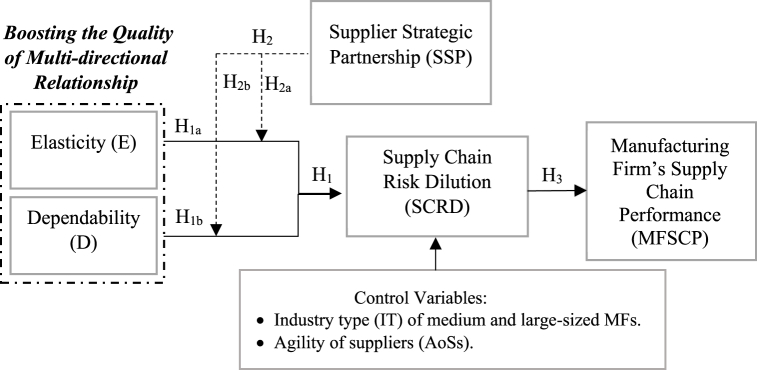
Study model (Source: developed by Author).

Within the context of elasticity and dependence theories, the availability of resources determines the level of reliance and dependence among supply chain participants [72,57,and82]]. Numerous studies view elasticity and dependence as complementary aspects from different points of view in the relationship of buyers-suppliers [47]. In this regard, research has evaluated the dependence of buyers and suppliers [55,38]. This study concentrated on the risk dilution provided by the supply partner and measured supplier involvement as the influential element in the relationship between QoMDR and SRD. All constructs used in this study were adopted from SCM literature [e.g. 4, 5, 22, 30], and linked together due to that there were associations between them in the previous studies and each construct has a significant impact and is linked to other constructs, that why used these constructs due to they are vital to MFSCP. The study model is illustrated in Fig. 1.

### 2.1 Quality of multi-directional relationship and supply risk dilution

The quality in the buyer-supplier linkage can be seen as a critical component in SC risk dilution, taking into account factors such as the trust-based, flexibility, elasticity, and commitment connections between the buyer and supplier, as well as the dynamic relationship founded on dependence [65]. Elasticity is regarded as a key factor in limiting exploitation among supply chain participants [21,24,58,68]. Furthermore, it is recognized as a key element in mitigating risks within the supply chain [19,71]. In intricate trading relationships, elasticity-based connections can reduce the likelihood of opportunistic behaviors [73,20]. Supply risks may arise when the buyer-supplier relationship is established, and an elasticity-based partnership can minimize supply risks for the buyer [74,67,64,49,and81]]. Moreover, the association and connection based on elasticity, flexibility, dependence, information sharing, and a problem-solving technique resulting from a strong buyer-supplier linkage can significantly influence adaptability to changes, the identification of appropriate solutions to organizational challenges, cost control, and increased revenue [65]. Thus, we suggested the following hypotheses.

H1a Elasticity in a buyer-supplier quality relationship has a direct significant positive effect on supply risk dilution in manufacturing firms in developing countries.

H1b Dependability in a buyer-supplier quality relationship has a direct significant positive effect on supply risk dilution in manufacturing firms in the developing countries.

### 2.2 Supply chain dilution, and supplier strategic partnership

Quick environmental changes can sometimes disrupt supply chain nodes, leading to non-positive commercial barriers and long-term risks for firms. As a result, many firms adopt various forms of elasticity, flexibility, and dependency to mitigate and dilute potential threats [75]. In today's hypercompetitive global markets, characterized by not long life cycles for goods, rapid IT tools advancement, and fluctuating customer expectations, firms must be able to cope with uncertain environments. Supply chain elasticity (SCE) has been divided into internal and external types in several prior research to ensure the continuity of logistical and operational processes [76]. described internal elasticity as a company's capability for quickly responding to any signals that come from the different markets when a supply disruption occurs while keeping a high level of preparedness for external emergencies. External elasticity, on the other hand, refers to the ongoing alignment of supply and demand between a company and its supply chain through dependable suppliers and end consumers. Numerous research studies have assessed the association between SCE and its impact on firm performance (Baz and Ruel, 2021, 53] [77]. explored and tested the effect of SC risk management on SCE, and [78] found that SCE significantly influences service firms' performance. However, some studies have concentrated on a one-dimensional side of SCE [49,79,75], while others have noted conceptual interference in the categorization of SCE's constructs [80]. For instance, elasticity, flexibility, responsiveness, and agility all reflect the ability of supply chains to react quickly and respond to market signals or potential threats at high speed, in contrast to descriptive and empirical research that solely concentrates on SCE's various dimensions [81].

Supplier strategic partnership (SSP) involves engaging suppliers in a more collaborative manner, allowing them to support firms in problem-solving and participating in new product development processes [82]. Some supply chain literature suggests that supplier participation and involvement in various firm activities can enhance performance and efficiency, positively affecting both financial and operational outcomes [39]. However, this may not always be the case, as few research have shown that formulating relationships with suppliers can have non-positive impacts on certain companies due to factors such as coordination costs, firm inertia, capacity variance, unclear objectives, and inconsistency [83]. These ambiguous findings suggest that the linkage between suppliers and companies might be indirect and obvious, with suppliers' roles possibly limited to supporting intermediate, emergency, and non-critical operations [84]. Some research emphasizes that formulating and coordinating with suppliers can provide companies with a channel for obtaining knowledge, facilitating the creation of diverse products with competitive advantages like product innovation [32,54]. Thus, a limited number of studies have explored the indirect effect of supplier collaboration and partnerships on companies' financial performance through cooperative product development and innovation efforts [85].

Supplier collaboration is a critical factor for growth and success in dynamic markets and for offering products in ever-changing environments. Firms that collaborate with dependable suppliers gain a competitive edge by enhancing their product development and addressing potential challenges [86]. As a result, partnering with suppliers improves performance, shortens product delivery time, and reduces coordination and logistical costs, ultimately boosting customer satisfaction [87]. Working with trustworthy, reliable, and supportive suppliers offers numerous benefits, such as access to planning and knowledge resources, a positive effect on shipping, operational elasticity, and various linkage of logistical activities. This collaboration also raises social responsibility levels by adopting sustainable strategies in changing environments [88]. [89] demonstrated that supplier collaboration strengthens firms' competitiveness by adopting and sharing various technologies, embracing technological advancements, implementing best practices, jointly developing new products, rapidly accessing markets, and flexibly responding to shifting demands. These collaborative efforts lead to increased financial benefits through various logistical operations, for example storing, packaging, delivery, handling of products, and procurements, which in turn reduce waste and enhance customer satisfaction. A study by Ref. [90] on an Indian manufacturing firm, in a developing country, revealed that collaboration with suppliers positively impacts supply chain performance, resource allocation, knowledge sharing, cost optimization, waste reduction or elimination, and management of reverse material flows and packaging [91].

### 2.3 The moderating impact of supplier strategic partnership on the quality of multi-directional relationship and supply risk dilution

The term strategic partnership, as defined by Ref. [23], refers to the number of traditional options available in a specific market location. From this perspective, having multiple alternatives within supply chain participants leads to increased involvement, collaboration, coordination, and cooperation [38,60]. Manufacturing companies experience leverage authority among other competitors due to their interdependence [5]. In essence, influence can be seen as the substance of numerous resources acquired [31]. [41] highlighted that weak supplier strategic partnership results in a wider range of options among purchasing firms, which in turn increases the risk of supply for the buyer. A company's relative authority position and involvement play crucial roles in risk assessment [53]. In situations of one-sided involvement, the more dependent party faces greater vulnerability. In case a supplier is more dependable on the buyer company, it makes greater efforts to improve its picture perceived by the buyer. To satisfy the buyer's attitudes and requests, the reliable supplier strives to provide the highest possible support [41]. Consequently, it leads that the supply risk will be decreased for purchasing companies.

Additionally [38], discussed the connection between linkage among SC partners and risk. They explained that opportunistic actions arise from the level of trust between parties and their propensity for self-interest, which results in a lack of confidence in the relationship. Consequently, each party's responsibilities and commitments are not fully fulfilled. Furthermore [[23], [92]], asserted that when a supplier depends on the purchasing company, it strives to meet all of the buyer's good feature requirements and criteria. Thus, it will lead to a positive impact on buyers' trust and increase the linkages between them.

Regarding the previous information, leads the formulation of the proposed hypotheses as follows.

H2a Supplier strategic partnership moderates the relationship between elasticity in a buyer-supplier quality relationship and supply risk dilution in manufacturing firms in the developing countries.

H2b Supplier strategic partnership moderates the relationship between dependability in a buyer-supplier quality relationship and supply risk dilution in manufacturing firms in the developing countries.

### 2.4 Supply risk dilution and manufacturing firms’ supply chain performance

The risks of supply can influence a company's outcomes, opportunity cost reduction, more efficient use of existing resources, and progress. Thus, mitigating and diluting supply risk is crucial for enhancing manufacturing companies' SCP. In this context [19], pointed out consequences such as unfavorable results, failing to satisfy customer requests or posing secure risks for consumers, reduced income and revenue due to consumer attrition, negative effects on goods durability and reliability, and good inability as the repercussions arising from supply risk. In the event of any interruptions in the supply of small and large manufacturing companies, it will delay the commitment to deliver orders to customers, which will reflect negatively on customer satisfaction, with revenues of about 30–40 % as losses from the total profits. Therefore, most manufacturing companies that operate in dynamic, rapidly changing environments have been forced to comprehensively review their policies and strategies to focus on several important elements so that they can be more flexible and faster in responding to customer requests in different markets and harmonize their operational processes and logistics facilities. So that, they are consistent with the requirements of these elements in order to mitigate the risks that these companies may face. From this standpoint, companies can obtain many sustainable competitive advantages, have a greater opportunity to survive in the cycle of intense competition in a volatile business environment, and increase their ability to deal with unexpected disturbances [93]. Therefore, the main objective of manufacturing companies and their SCs is the necessity of providing RMs and production resources minimizing costs, and securing them at the specified period and in the appropriate place in order to quickly face the demands of consumers. Several factors are negatively affected in the event that the total costs are reduced from the minimum, for example, the on-time delivery of requested customers' orders, which is related to the predetermined time limit [94,95]. The study of [43] emphasized that mitigation of SC risks has been reduced by taking into account several other factors such as the emergence of local, regional, or even global financial crises, economic stagnation, and recession, the emergence of new competitors, the emergence of new services and technologies, all of which represent macroeconomic challenges. The research concentrated on the manufacturing sector taking into consideration the SCs' effectiveness and efficiency as well as reducing the risks that it may face in dynamic markets and business environments with volatile demands. Therefore, in order to reach high efficiency in the performance of supply chains of manufacturing companies, it was necessary to focus on the need for continuous improvement in their operational and logistical operations, in addition to relying on a system to reduce internal and external risks related to supply chains [65]. study focused on both factors (i.e., efficiency, and effectiveness) were investigated and examined in the manufacturing companies. Therefore, the H_3_ hypothesis is raised as follows.

H3 Supply risk dilution has a direct significant positive effect on manufacturing firms' supply chain performance in developing countries.

### 2.5 The mediating effect of supply chain dilution in the relationship between the quality of multi-directional relationships and manufacturing firms’ supply chain performance

In [96] study, he presented a model that includes the structure of requests or services for customers, in which he asserts that it is not the same for all manufacturing firms, which can be and include functional or even creative elements. Therefore, he considered flexibility in the SCs of manufacturing companies as “efficient” or “responsive” characteristics and elements and then presented in his study the appropriate type according to the nature and structure of the firm and the market in which it operates. Therefore, the [96] model is considered a basic model in the strategy of manufacturing firms in selecting their suppliers and building relationships with them, noting that most of the products at present have a short life cycle. In addition to the requests, being constantly changing and volatile, and the customer is looking forward to obtaining products of high quality and diverse and unique characteristics. Therefore, this model is considered a roadmap in building a corporate strategy that can be relied upon so that supply chains are more elastic, flexible, and responsive to markets to reduce inventory levels, speed, and agility in sending orders to customers at their specified times. In the SCM literature, there were many empirical studies, but they were only partial and on specific factors, including the SC risk mitigation factor, which has a large and important impact and plays an active role in the performance and efficiency of the supply chain, especially in manufacturing companies [22]. The focus on the concepts of elasticity and dependability in most manufacturing companies has become a significant factor in order to obtain multiple comparative advantages and achieving high levels of sustainability in the SCPs in rapidly changing and volatile business environments [5]. Either some previous studies dealt with the risk reduction factors in SCs, the performance of supply chains, or the speed of response, separately or together to fulfill diversified customer orders [23].

There is much prior research focused on the importance of flexibility in the various operational and logistical processes in manufacturing firms [94], while a number of other studies indicated that there are still some differences in properties in order to achieve this [4]. The study of [18] refers to the compilation of these concepts and terminology in what is called "legitimacy", which would raise the ability to manufacture companies and their SCs to fast responding to customers, in addition to the dynamism of dealing with market fluctuations with high efficiency, which leads to achieving sustainability in flexible performance. Although most manufacturing firms currently benefit from the feature of adopting and applying elasticity and dependability, a number of firms still need to increase thinking and clarification on the mechanisms of their work in their various internal or external operational and logistical activities. From this standpoint, it is necessary to define the scope of their work and how to apply them with clear procedures when building their strategies to achieve real comparative advantages and obtain sustainable performance (i.e. SC) in order to quickly access different markets, and enhance performance levels. From this scenario, the following hypotheses were formed.

H4a SC risk dilution mediates the linkage between elasticity in a buyer-supplier quality relationship and manufacturing companies' SCP in developing countries.

H4b SC risk dilution mediates the linkage between dependability in a buyer-supplier quality relationship and manufacturing companies' SCP in developing countries.

This study is considered a moderated-mediation scenario applied model, which tries to investigate the impact of SSP on the linkage between QoMDR and SCRD constructs [97]. Then tries to investigate how SCRD construct can affect the relationship between QoMDR and MFSCP as well; and due to that, all adopted constructs are vital and have a significant impact on MFSCP as mentioned in the SCM literature. The industry sector (i.e., manufacturing) depends mainly on managing effectively logistical activates within the SC to guarantee to provide the required quantities of RMs, items, components, and semi-finished goods to the right location, at the right time, with the right quantities, and at a reasonable cost. Therefore, elasticity and dependability features will boost the quality of the multi-directional relationship via building vital strategic supplier relationships in order to mitigate SC risks that might support manufacturing companies in improving the logistical operations of their SCP.

The rationale or justification for doing this research study was gleaned from the existing literature on the subject. We conducted a thorough literature survey and identified gaps in the current literature, which was the best way to go ahead with building the study model and then formulate the required hypotheses that are related to the main dimensions that were included in the model to be tested. Thus, we did not repeat what others have already stated and our perspective did not emerge from thin air. For this study, we have actually created, developed, and formed testable working hypotheses based on the recommendations of some research studies mentioned in the literature to fill the gap in knowledge particularly in the process of adopting, forming, and building strong relationships between business partners in the management of corporate SCs. Through achieving, common and mutual interests among them and their preference for not working individually and separately due to recently the intensified competition between companies. This was the first step towards conducting and applying our current research study, especially in developing countries that are located in the Middle East region, in order to prove or refute the hypotheses formulated in this study. We considered online surveys as a primary data collection tool to help us generate and formulate hypotheses according to the conceptual research model of the study. After that, appropriate techniques were selected to analyze and test the proposed hypotheses. Moreover, we have already prepared and formulated appropriate hypotheses usually based on previous relevant evidence-based studies, which were mainly based on relational view theory (RVT) (i.e. trust, commitment, flexibility, agility, green, elasticity, responsiveness, dependability, collaboration, confidence, etc.).

The hypotheses that were mentioned and formulated in this study depend on the literature that is related to the subject of the study and on evidence-based based-logical justifications, as previous studies were well received by the scientific and professional community in studying relationships and their importance in SCs in light of the intense competition in the business world that is very volatile and changing. The research study tested a specific number of hypotheses that had already been formulated based on the study model that was built. These hypotheses were carefully planned to ensure appropriate methodology, appropriate statistical analysis, and test power. Therefore, the study hypotheses are the starting point for examining the ideas contained in our conceptual framework, which helps in verifying our results, the data collected, and/or other previous study results.

This paper concentrates on the significance of adopting and using elasticity, and dependability features through the quality of the multi-directional relationship via reliable strategic partnerships with multiple partners within the SC network that includes several manufacturing companies in general in order to mitigate SC risks and enhance their SCP, the emerging and developing countries as sources of supply in particular. Thus, this paper tries to contribute particularly in the field of boosting the quality of multi-directional relationships and their adoption and implementations in some of the third world countries (i.e., emerging, and developing) as illustrated in the below main key points.

➢ Through a quick survey of previous studies related to the subject of the study, none of these studies addressed any of the factors and dimensions that were included in the proposed study model combined with each other in one model, but rather some of these factors were studied individually or partially together. Such as enhancing the quality of multi-directional relationships through flexibility and dependability features. Addition to studying the effect of the strategic partnership factor with suppliers as a moderating factor and focusing on the effect of the SC risk mitigation factor as a mediating factor in order to enhance the performance of the supply chain of manufacturing companies, especially in countries located in the Middle East region as developing and emerging countries.
➢ The majority of the scanned previous empirical studies that are related to the study topic were applied to developed countries, and there were no empirical quantitative studies on emerging or developing countries, especially countries located in the Middle East region.

## 3 Research methodology

The next sub-heading will present the data collection process, instrument tool used, population, and sample.

### 3.1 Data collection process and sampling technique

This study adopted positivism and realism research philosophies using quantitative methodology approach-deductive hypothesis testing, further, the researchers used the descriptive analytical approach. Using quantitative methodology does not enable the researcher to intervene directly or indirectly by influencing the group participating in filling out the questionnaire; rather they have complete freedom to fill the survey at any time to express their opinions without any pressure or influence [98]. The online survey served as the data collection method using a simple random sampling technique for testing the study hypotheses of small to large-sized manufacturing companies in the Middle East region as emerging and developing countries. All data gathered was only used for research purposes with a high confidentiality process as mentioned in the survey cover letter. The results of this study will be available and can be provided for the targeted respondents upon their request. Participants were reminded to fill out the study questionnaire by sending another email two weeks after sending the first email to motivate and encourage them to fill out the questionnaire attach by cover letter, then as a follow-up process, after two weeks we did a kind reminder for them. The population of this study represents small to large-sized manufacturing firms listed on the Stock Exchange Markets (SEMs) in Jordan (36), Turkey (42), King Saudi Arabia (48), and Egypt (30). The data collection took place in the period March until July 2022 using Statistical Package for the Social Sciences (SPSS v.28.0.1). The sample of this study targeted the respondents that included top and mid-level professionals, such as chief executive officers, presidents, senior's managers, joiner supervisors, production and operational managers, IT managers, logistical managers, and distribution directors from 743 firms that participated in the online questionnaire. 156 received appropriate and useable surveys out of 743 were received, resulting in a 21 % response rate, as shown in Table 2 [99]. indicated that a good and acceptable online survey response rate ranges between 5 % and 30 %, while an excellent response rate if it is >30 %. The responding firms spanned various industrial sectors, including medical and pharmaceutical, paper, IT, furniture and wood, textile, and rubber and plastic firms, with sizes ranging from small to large-sized manufacturing companies (i.e., 51 employees and above).

**Table 2:** Participating manufacturing firms’ characteristics (Source: developed by Author).

Manufacturing Firms Sectors	#. of Manufacturing Firms Listed in SEs (Jordan, Turkey, King Saudi Arabia, and Egypt)	#. of Participating Manufacturing Firms	Frequency (%)
Electrical/IT Manufacturing	106	26	16.6
Chemical/Cosmetics Production	101	18	11.5
Pharmaceutical/Medical	91	20	12.8
Paper/Tissues/Packing Processing	134	14	9.00
Textile/Clothes Manufacturing	76	24	15.4
Plastic/Rubber Processes	47	17	10.9
Food and Beverage Processing	131	21	13.5
Furniture/Wood Manufacturing	57	16	10.2
**Total**	**743**	**156**	**100 %**

### 3.2 Constructs’ correlation and normality distribution test of data

The half-split method was applied for finding the Pearson correlation coefficient between the mean of odd-ranked and even-ranked items for each construct in the survey. Then, we corrected the Pearson correlation coefficient using the corrected Spearman-Brown correlation coefficient. The normal range for the corrected correlation coefficient is 0.0 to +1.0. Additionally, we performed a sample Kolmogorov-Smirnov test to determine whether the study questionnaire data follow a normal distribution. This test is considered necessary for hypothesis testing because most parametric tests describe a normal distribution of data [100]. Table 3 summarizes the results that are obtained by the split-half coefficient method and one sample Kolmogrov-Smirnov test.

**Table 3:** Summarizes the results that are obtained by the split-half coefficient method and one sample Kolmogorov-Smirnov test. (Source: developed by Author).

Construct	Split-Half Coefficient Method		One Sample Kolmogorov-Smirnov Test	
	Pearson-correlation	Spearman coefficient	Z-value	Sig.
**QoMDR**	0.729	0.904	0.141	0.201
**SCRD**	0.746	0.931	0.156	0.252
**SSP**	0.851	0.940	0.162	0.190
**MFSCP**	0.753	0.862	0.147	0.183

A common bias method (CMB) was employed to mitigate the effects of non-response bias, as suggested by Ref. [101]. Consequently, a comparison between two sets was applied in two periods/phases (i.e., pre, and post-phase), and the respondents' responses were conducted to assess the non-response bias effect. In the early phase, 76 respondents participated, while 79 participated in the late phase. The comparison results showed no vital differences between the two sets in two different phases, alleviating concerns about non-response bias. This study found no notable differences between the two sets across the selected criteria in the mentioned phases. Additionally, as shown in Table 6, Harman's single-factor test was used, with the results also indicating a non-response bias effect [[102], [103]]. As highlighted by Ref. [103] if a single factor or the primary one accounts for a significant portion of the total variance, the Factor Analysis Test (FAT) results showed that 86% of the total variance was identified after combining the four constructs illustrated in the study model. For other verification and as a double-check test, as recommended by Ref. [101], a Single Factor Analysis Test (CFA) was also performed. All constructs and elements were loaded onto a single factor, and the results indicated a poor fit with a relative Chi-square value of 28.43, NNFI of 0.802, CFI of 0.835, and RMSEA of 0.576. All prior findings demonstrate that CMB is not a significant aspect of this research.

### 3.3 Measures used in the study

In this section, we describe the measures used for the research dimensions. We derived and adopted an eight-item scale for buyer-supplier quality of multi-directional relationship (QoMDR), split into two dimensions (i.e., elasticity (E) and dependability (D)) from the [35] study. To measure the elasticity in the buyer-supplier relationship dimension, a four-item scale was employed, and another four-item scale was used for gauging the dependency aspect of relationship quality. A five-point Likert scale was utilized in this study, with (1) representing "strongly disagree" and (5) signifying "strongly agree" for measuring the relationship quality dimensions. We adapted and refined three elements from Ref. [25] study to assess supplier strategic partnership (SSP) by applying a five-point Likert scale. To evaluate the risk dilution dimension, we adopted, refined, and employed six elements from Ref. [23] study, also using a five-point Likert scale, 1 represents "strongly disagree", while, 5: represents "strongly agree." Lastly, from the [59] study, we adopted, refined, and used a ten-item scale to measure the MFSCP dimension, applying a five-point Likert scale ranging from 1 to 5, as implemented by the [59] study. To reach a high level of verification of survey content validity, the questionnaire was presented to a number of 4 academics and 2 practitioners related to the subject of the study, who have long experience and knowledge in the subject of this study in order to conduct a review of all the contents of the study tool and the method of measurement and to make any comments from them as feedback. The necessary amendments were made based on their comments, such as repetitive and/or ambiguous items, or deleting them from the measurement survey tool before launching a large-scale questionnaire, bearing in mind that the questionnaire was in English at the time of collecting the initial data.

Additionally, the firm's number of years working in the market and the number of essential, dependable suppliers are considered control variables in this study's proposed model. Through scanning the related literature, we observed that numerous research, for example [18,22], and [104], have illustrated that the few potential and reliable strategic partnership suppliers might influence buyers' authority and tangible potential supply risk [53]. contended that much of trustworthy suppliers suggest that the buyer has the ability to transfer to another supplier as another option scenario. Table 4 summarizes the primary statistical analysis and displays the scales used in this study.

**Table 4:** Summarizing the primary statistical analysis of the study (Source: developed by Author).

Variables (α)	Items	Coding	Loading	*Composite reliability*	AVE
Elasticity (α = 0.91)	In the case that any problems arise, the suppliers are obliged to cooperate with us in solving them (i.e., shipping interruption).	E1	0.79	0.91	0.741
	Suppliers are always in continuous cooperation with us	E2	0.77		
	False allegations, if any, are made by our suppliers	E3	0.79		
	Our suppliers are always under their promises.	E4	0.86		
Dependability (α = 0.83)	We rely heavily on our suppliers for our distribution channels.	D1	0.72	0.85	0.722
	There is a weak link in our relationship with our suppliers.	D2	0.91		
	There is high cooperation between our firm and suppliers.	D3	0.87		
	We are always working on strengthening business relationships with our suppliers.	D4*	0.31		
Supplier Strategic Partnership (α = 0.88)	There are other competitors in our field of business within our commercial areas.	SSP1	0.84	0.89	0.775
	In the case that our services are dispensed with another company, the suppliers bear some of the total costs with us.	SSP2	0.87		
	If some of the sales and profits of our company are replaced, the suppliers will bear the part of the difficulties.	SSP3	0.80		
Supply chain risk Dilution (α = 0.92)	Most suppliers adhere to our specifications.	SCRD1	0.77	0.87	0.692
	Our suppliers have a relatively high commitment to shipping requirements and adherence to specifications and instructions (i.e., short lead-time).	SCRD2	0.86		
	The commitment of our suppliers is relatively high especially with regard to meeting our volume orders.	SCRD3	0.74		
	Suppliers in general, are committed in most cases to fulfilling our orders.	SCRD4	0.89		
	Suppliers provide us with different orders on time without any delay.	SCRD5*	0.38		
	Our suppliers can ship our orders based on our requirements in most cases.	SCRD6	0.81		
Manufacturing Firm's Supply Chain Performance (α = 0.87)	Most of our logistics-related costs are reduced through our dependable supply chain.	MFSCP1	0.85	0.81	0.753
	Logistics operations and activities are reduced through the efficiency of our supply chain operations.	MFSCP2	0.77		
	Warehousing costs are reduced through the efficiency of our supply chain operations.	MFSCP3*	0.40		
	Our SCPs reduce inventory costs.	MFSCP4	0.86		
	Because of adopting effective projects in operational and logistical operations, large profits and returns are achieved for the company.	MFSCP5	0.88		
	The firm's order fill rates are increased due to our effective SCPs.	MFSCP6	0.78		
	The firm's inventory turns are increased due to our effective SCPs.	MFSCP7	0.84		
	The firm's safety stocks are reduced due to our effective SCPs.	MFSCP8	0.76		
	Due to our effective SCPs, we try to reduce the firm's inventory obsolescence.	MFSCP9	0.89		
	Due to our effective SCPs, the firm tries to reduce products warranty and guarantee claims.	MFSCP10*	0.42		

Note(s): * Removed item; item loadings after deleting values < 0.5.

To evaluate the study hypotheses that are formulated in the model, the partial least squares approach employed Smart PLS-4 (version 3.2.6). We utilized the R-square to gauge the explained variance. According to the [105] study, the threshold values for R^2^ analysis were set, with the following findings: R^2^ small = 0.002, R^2^ medium = 0.23, and R^2^ large = 0.321. The findings are illustrated in Table 5.

**Table 5:** Summarizes the Inner-Model Analysis results (Source: developed by Author).

*Main Variables*	*R*^*2*^	*Adjusted R*^*2*^	Effect*size (f*^*2*^*)*
Elasticity (E)			0.345
Dependability (D)			0.072
Supplier Strategic Partnership (SSP)			0.083
Supply Chain Risk Dilution (SCRD)	0.686	0.745	0.458
Manufacturing Firm's Supply Chain Performance (MFSCP)	0.231	0.226

**Table 6:** Discriminant validity and collinearity results using the Heterotrait-Monottrait ratio of correlations (HTMT) (Source: Developed by Author).

Constructs	1	2	3	4
1. Quality of Multi-directional Relationship (QoMDR)	0.863			
2. Supply Chain Risk Dilution (SCRD)	0.836	0.887		
3. Supplier Strategic Partnership (SSP)	0.761	0.791	0.847	
4. Manufacturing Firm's Supply Chain Performance (MFSCP)	0.740	0.748	0.763	0.879

## 4 Analysis of the study

The next sub-heading will illustrate the reliability, validity, and descriptive statistics used.

### 4.1 Reliability, validity, and descriptive statistics

The Heterotrait-Monottrait (HTMT) correlation method was used to examine and test the discriminant validity and collinearity of all dimensions that were included in the proposed model for the study. The findings indicated that all values showed a value of <0.9, and this indicates that there are no collinear relationships between the dimensions according to a study of [106]. Table 6 shows the findings of collinearity. The findings also showed that discriminant validity was achieved as shown in the values included in Table 4, all of which are also <0.9.

### 4.2 Study results

The Confirmatory Factor Analysis (CFA) technique was conducted in Smart PLS-4 to filter the items, validity (V), and reliability (R) for all measurement scales in the model. After eliminating three items (D4 = 0.31, SCRD5 = 0.38, MFSCP3 = 0.40, and MFSCP10 = 0.42) with weak performance loading <0.5, a reasonable fit was achieved, with RMSEA = 0.092, CFI = 0.91, IFI = 0.94, and χ^2^ = 464.58; df = 247 (the rate of $\chi^{2}$ to degrees of freedom ratio is satisfactory, equal to 2.05). Table 5 provides a summary of all measurement analyses for the model, encompassing item loading (ILs), composite reliabilities (CRs), average variance extracted (AVE), and Cronbach's alpha (α). It should be noted that the CR, AVE, and α for each construct in the study model exceed the threshold values, with scores of 0.7, 0.5, and 0.6, respectively [106]. Additionally, convergent validity was confirmed, as all item loadings were > 0.5 and statistically significant at < 0.05 level.

### 4.3 Hypotheses testing and discussion of study results

Manufacturing companies that are part of the industrial sector in the Middle East region have an active role in economic development through production capabilities in the target countries included in the study through effective management that includes safe access to supplies such as raw materials, components, and finished goods at the right time, and in sufficient quantities and at a reasonable cost. Therefore, adopting certain characteristics such as flexibility, elasticity, dependability, and reliability in its relationships with suppliers and business partners helps these companies increase their effectiveness and efficiency. The experimental study [5] indicated in its findings the importance of elasticity, as it was found to have a positive impact on improving the performance of SC in its supply of materials and resources to manufacturing companies, through building strong and solid strategic partnerships with suppliers and relying on them for the success of their production and operational processes as well as their logistical activities.

This study assesses the effect of elasticity and dependability as features of quality buyer-supplier relationships on SC risk dilution directly. Furthermore, examines the moderating impact of SSP on the linkage between the quality buyer-supplier relationship and supply chain risk dilution, and the mediating effect of SC dilution in the linkage between quality buyer-supplier relationship and MFSCP. Thus, this research focuses on the significant role of elasticity and dependability elements as features for local and/or global suppliers for manufacturing firms in their several functions and operational activities. In addition to the existence of a risk reduction system in their supply chains particularly in the Middle East region as an important source of supply chains to leverage their efficiency and increase their effectiveness. Therefore, this study was conducted due to its importance in bridging the gap in the SCM literature in the areas of quality of multi-directional relationships with local and global suppliers and even with trading partners through such features as elasticity and dependability, reducing supply chain risks, strategic partnership with suppliers, and its effect on increasing the SCP of manufacturing companies in the Middle East emerging and developing countries, this is more accurately illustrated by the following points.

➢ The significant factors included in this paper were not obtained by previous studies in the literature for concentrating on elasticity and dependability as vital features of suppliers and other partners as comparative advantages through clustering them in one package model and their impacts on dilution SC risks in emerging and developing countries located in the Middle East region.
➢ Many prior researches that focused on this vital topic were conducted in developed countries, while a few studies were performed and rarely used in developing and emerging countries.

**Fig. 2:**
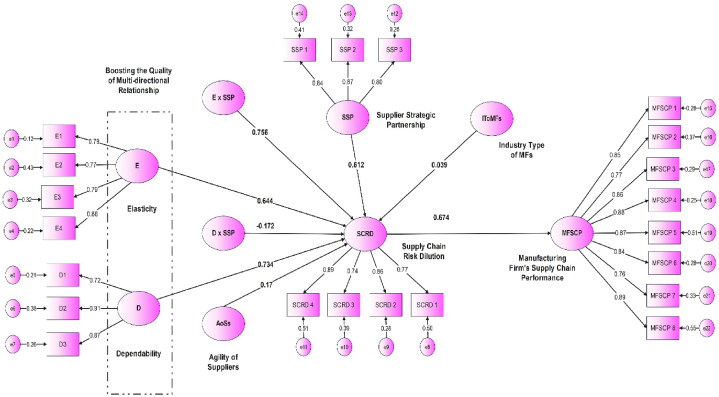
Study model with all included constructs standardized coefficients and t-values) (* significant at 0.05 level) (Source: developed by Author).

This paper has assessed the causal associations of the proposed hypotheses that were obtained in the study model using structural equation modeling/PLS-4. This program was performed to ensure the associations between QoMDR, SSP, SRD, and MFSCP dimensions. The findings as explained in Table 7 illustrate that all hypotheses were supported except H2b. Table 7, Table 8 illustrate the findings of the direct and indirect paths. In addition, it mentions the direct path coefficient for the E-SCRD relationship which is (β = 0.644, t = 5.376, p = 0.001 < 0.05). Thus, it illustrates that there is a direct positive and statistically significant effect. Furthermore, the H1a hypothesis explains that there is an effect between the elasticity (E) and SCRD factors, which is strong and has a direct positive significantly, which means that we already have to accept the proposed hypothesis. This formulated hypothesis (i.e., H1a) says that elasticity in a buyer-supplier quality relationship has a direct and positive impact on supply risk dilution in manufacturing companies in the countries that were targeted in the Middle East region as emerging and developing countries. Thus, this finding is consistent with pieces of works previously published in the SCM field, for example, findings mentioned by Refs. [67,22,3], and [71]. This suggests that when key suppliers avoid making false claims, maintain trustworthy promises, and are honest about emerging issues, buyer firms can effectively reduce their supply risk and disruption. Fig. 2 displays the study model with mentioned of all constructs included, standardized coefficients, and t-values. This shows that manufacturing companies that use and adopt elastic, flexible, and reliable suppliers as features of their partners try to ignore failure claims, do their process and functions with reliable mentioned, displayed, and announced criteria as committed upon, and are active to solve any problem(s) that might be happening. This issue will encourage the buyer companies to dilute their supply risk and minimize the probability to go into any interruption of supply.

**Table 7:** Outcomes of direct and indirect relationships (Source: developed by Author).

*Hypothesis/Path*	β*-value*	*Std. error*	*t-statistic*	*p-value*	*95 % Confidence interval*	*Remark*
H1a:E-SCRD	0.644	0.067	5.376	0.001	{0.246, 0.438}	Accepted
H1b:D-SCRD	0.734	0.036	6.350	0.026	{0.445, 0.670}	Accepted
H2a:E*SSP*SCRD	0.756	0.078	4.432	0.002	{0.067. 0.358}	Accepted
H2b:D*SSP*SCRD	−0.172	0.047	0.431	0.361	{-0.331, 0.239}	Not accepted
H3:SRD-MFSCP	0.674	0.043	4.722	0.031	{0.347, 0.738}	Accepted
H4a:E*SCRD*MFSCP	0.472	0.042	5.412	0.020	{0.061, 0.473}	Accepted
H4b:D*SCRD*MFSCP	0.681	0.052	4.162	0.034	{0.239, 0.442}	Accepted

**Table 8:** The regression analysis on the dependent variables^a^ (Source: developed by Author).

Model	Variable/Constant	B	t	Sig.	Result
*1*	(Constant)	3.834	37.501	<.001	–
	Control variable	−.001	−.013	.990	No
*2*	(Constant)	.090	.422	.673	–
	Control variables	.080	1.717	.087	No
	Industry type (IT) of medium and large-sized MFs.	.039	.741	.459	No
	Agility of suppliers (AoSs).	.170	3.159	.002	Yes

a Dependent variable SCRD.

Furthermore, the findings show that a direct path between dependable (D) in a buyer-supplier quality relationship and supply chain risk dilution (SCRD) has a direct positive and statistically significant impact (β = 0.734, t = 6.350, p = 0.026 < 0.05). Thus, the H1b hypothesis explains that dependability in a buyer-supplier quality relationship has a direct and positively significant impact on supply risk dilution in manufacturing firms in the emerging countries that are located in the Middle East region, which is also supported. This finding is in line with a number of published papers in the literature, such as conducted by Refs. [23,19,65], and [42], which emphasize the crucial role of dependable and reliable suppliers with manufacturing firms in distribution channels, strong collaboration with their suppliers and other partners within the SC, and improvement of business relationships with suppliers' strategic partnerships alike. In brief, the extent of supply risk a buyer may be willing to tolerate relies on several elements, for example, the dependable supplier's not capable to meet the buyer's quality criteria and designation requirements, shipping not arrived on time, the buyer's existing elasticity in the supplier, and the supplier's dependability in fulfilling manufacturing firms' commitments and their orders. However, the buyer will be more trusted with the supplier's dependability in closing its customers' order commitments. Thus, the findings show that the direct path between SCRD and MFSCP association has a direct positive relation and statistically significant impact (β = 0.674, t = 4.722, p = 0.031 < 0.05). Therefore, the H3 hypothesis, says that supply risk dilution has a direct positive significant impact on manufacturing firms' SCP in the emerging and developing countries, which is also confirmed. Thus, this finding is very close and in line with [12], and [42] studies.

In regards to the moderated-mediated model test, we went in two sceneries as follows: firstly, the mediating indirect impact of SCRD on the association between QoMDR (i.e., E and D elements) and the MFSCP constructs was tested and calculated. We used a 95 % confidence interval if it has (0.0) exists within 2 intervals; this shows that there is no correlation between the two elements with a non-statistical significant effect of the mediation factor as recommended by Ref. [107]. The findings as explained in Table 7 shows that all confidence intervals do not have a (0.0) within their ranges (H4a and H4b), this illustrates that the SCRD construct significantly mediates the association between E: (β = 0.472, t = 5.412, p = 0.020 < 0.05), D: (β = 0.681, t = 4.162, p = 0.034 < 0.05) and MFSCP. Hence, as we proposed H4a and H4b hypotheses, it says that SCRD mediates the association between elasticity and dependability in a buyer-supplier quality relationship and manufacturing companies’ SCP in the countries that are located in the Middle East region as emerging and developing countries. Therefore, SCRD significantly mediates the association between QoMDR and MFSCP (i.e., E: β = 0.472, t = 5.412, *p=*0.020 < 0.05; D: β = 0.681, t = 4162, p = 0.043 < 0.05) as well.

Secondly, we mentioned in our model that SSP moderates the associations between E, D, and SCRD. The findings show that there is a moderating influence value for E: (β = 0.756, t = 4.432, p = 0.002 < 0.05) on SCRD, and D: (β = −0.172, t = 0.431, p = 0.361 > 0.05) on SCRD. Hence, as we proposed in H_2a_ and H_2b_, it says that supplier strategic partnership moderates the relationships between elasticity and dependability in a buyer-supplier quality relationship, and SC risk dilution in manufacturing companies in the countries that are located in the Middle East region as emerging and developing countries. This shows that H_2a_ is confirmed, while H_2b_ is not. Additionally, R^2^ was estimated to examine the explanatory power of all constructs included in the proposed model. This explains the level of variance that is interpreted by the external dimensions in internal dimensions. It appears that the value of R^2^ was 0.745 %. This finding shows that the variance in the internal construct MFSCP is high if E, SCRD, and SSP are boosted to interpret 74.5 % of the variance in MFSCP. The value of R^2^ for the association between D and SCRD was 23.1 %. This result aligns with the findings of [5,23], and [59], which suggest that SCRD can enhance manufacturing firms’ performance throughout the entire supply chain network.

**Fig. 3:**
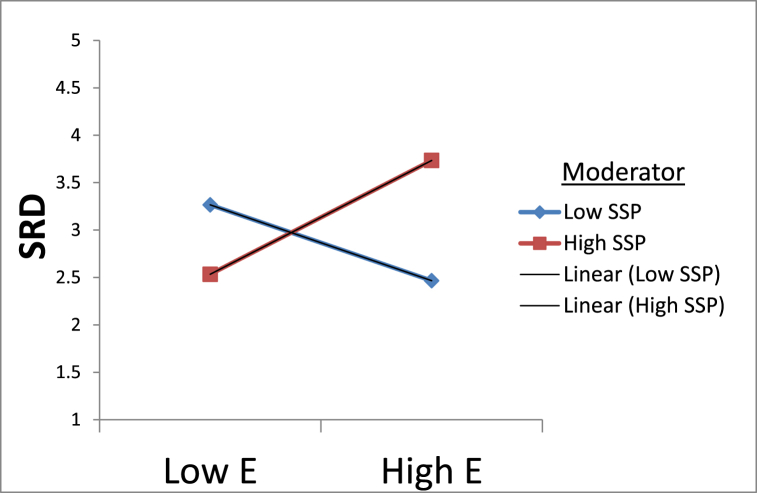
SI moderates the relationship between E and SRD (Source: developed by Author).

**Fig. 4:**
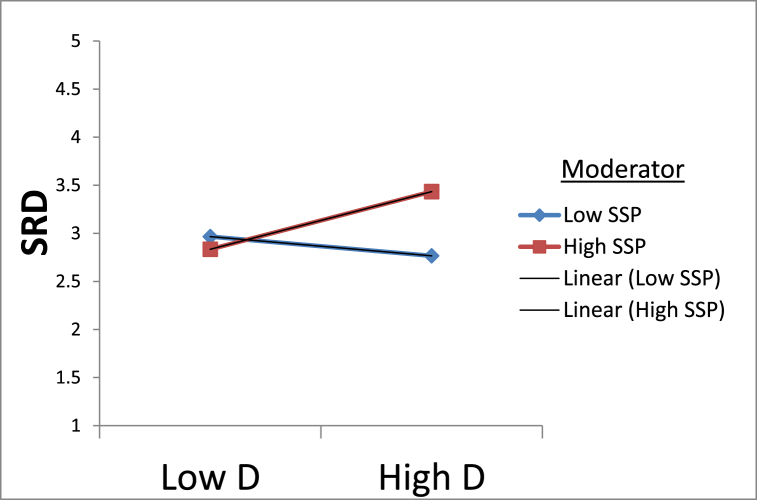
SI moderates the relationship between D and SRD (Source: developed by Author).

Fig. 3, Fig. 4 show the associations and paths that link the main dimensions to each other within the study model, showing estimates of the inner paths between the external constructs and the internal structure, including the enhancing and boosting role that plays the important and decisive factor SSP in each of E, D, and SCRD, respectively.

### 4.4 Control variable effect

The regression analysis was performed to estimate the effect of the QoMDR on the SCRD with the role of the control variable. Table 7 shows the findings of the regression analysis. Table 8 illustrates the regression analysis findings on the mediated role.

Two models were used to explain the behavior of the variables under study. The first model shows the role of the control variables on the dependent variable (SCRD), while the second variable explains the behavior of all the variables including the two dependents (i.e., E and D). By looking at the first model, it is noted that the unstandardized coefficient of the control variable was low (β = −0.001), moreover the significant value of the variable was insignificant (Sig. = 0.990 > 0.05) since it is greater than 0.05 [108]. This indicates that there is no clear impact of the QoMDR on the SCRD in the MFs'. In the second model, all the variables were entered to assess their impact on the dependent variable/SCRD. The analysis revealed only a strong significant impact of the several key agility of suppliers on the SCRD. (Sig. = 0.0020 < 0.05) However, the same model does not explain a significant effect of the control variable and through industry type of medium and large-sized MFs’ variables on the SCRD, since their (Sig. = 459 > 0.05) [108]. The second model justifies the results interpreted by the first model.

## 5 Conclusion

This study seeks to enlarge upon published papers by assessing the impact of the quality of multi-directional association (QoMDR) between buyers and suppliers on manufacturing companies' SCP through supply risk dilution (for example, [50,60,46,and82]]. Recent studies have highlighted that the expense of acquiring a new consumer is 5 times more than retaining existing ones, explaining why manufacturing firms continually strive to foster building high levels of quality associations with their customers [41]. As such, many studies have recognized elasticity and dependability as key components of QoMDR between suppliers and buyers in the supply chain [24,23,and85]]. Consequently, this research focuses on the primary constructs in the proposed model to analyze and examine the between supplier and buyer QoMDR and manufacturing firms’ supply chain performance, in addition to the moderating impacts of supplier strategic partnership and supply risk dilution on this association.

To achieve the study objective, we analyzed the association between multiple components of QoMDR and SCRD. Subsequently, the collaborative influence of QoMDR elements and SSP on SRD was explored. Finally, the role of SR dilution on MFSCP was investigated and evaluated. The findings of the H1a hypothesis support previous research by Refs. [67,19,65], and [20], demonstrating the impact of confidence and associations relied on the absence of illegal benefits in diluting SR. Furthermore, a fundamental and direct association between elasticity and SCRD is consistent with prior study findings [e.g., 21, 24, 84, and 104].

Moreover, the findings reveal that there is a direct and positive moderating effect of SSP on the association between dependability and SCRD. Conversely, the interdependence effect on SCRD resulting from the integration of SSP with dependability was not supported. Furthermore, this study corroborates the assertions made by Refs. [67,59], and [19] regarding the link between SCRD and MFSCP. Based on the results, particularly the moderating impact of supplier strategic partnership, it is recommended that future research investigate this relationship over a more extended time.

### 5.1 Theoretical and managerial implications

The findings of this study have shown many significant benefits and theoretical implications that should be taken into consideration by managers and decision-makers as well as industrial practitioners and from which they may benefit. First, most companies have resorted to building strong relationships and creating multiple partnerships in the form of effective economic poles with commercial partners and approved suppliers who have a reputation and high performance and have unique features such as reliability, dependability, high quality, elasticity, and flexibility. This has increased over the last two decades, especially in light of the environment, highly competitive and rapidly changing business. Therefore, it pushed manufacturing companies towards adopting and using various and multiple relationships that depend primarily on high quality with external parties and partners to achieve an integrated alliance in order to achieve joint and mutual benefits in order to increase their capabilities in providing their customers with the required products and high-quality services, in addition to reducing waste of resources, and the sources used, reducing costs, as well as increasing their competitive level. Secondly, the findings of this study focused on two main key elements for building multiple high-quality relationships by manufacturing companies with trading partners and suppliers, namely elasticity and dependability. If these important characteristics are adopted, they will have a positive and decisive impact in achieving better performance and obtaining many competitive advantages, as indicated by several previous studies such as [[13], [109],^19^], and [25,10]. Although there are many manufacturing companies in various manufacturing sectors that realize the importance of these elements, especially working in light of intense competition and unstable and constantly fluctuating business environments, they are still hesitant to adopt and activate these features with their business partners and suppliers, especially in private supply chains. However, there are a number of other manufacturing companies that do not know how to implement these features partially and/or completely, or they can focus on only one aspect and ignore other aspects that may have a significant impact on building relationships. Therefore, this study came to show its important findings to decision-makers and managers at various levels of management and industrial practitioners in manufacturing companies on the importance of building partnerships that depend on the quality of relationships so that they are multi-directional and multi-sourced in order to improve mutual benefit with business partners, reduce the risks of interruption of supplies and waste of resources and time. This negatively affects customer satisfaction levels and company performance in general. Third, the findings of this study provide a set of special measurement tools to measure key elements in building multiple relationships of high quality by using elasticity, reliability, and dependability as their characteristics. Additionally, reducing the risks of supply chains for industrial companies as well as building long-term strategic partnerships with certified and dependable suppliers of high quality, high reputation, and performance. Despite the presence of several other different measurement tools that were used and applied by previous studies that addressed some aspects of this study. This study came to give researchers and those interested in the research and academic fields the ability to use this measurement and apply it in their own companies to directly evaluate the results of the quality of multiple relationships in order to reduce the risks of their supply chains. Furthermore, increases the level of awareness and understanding of the impact of the strategic partnership of suppliers on the performance of their companies [26]. Fourth, the findings of this study ensured the key roles of QoMDR and SRD in enhancing and improving MFSCP.

This study presents several managerial implications, as follows: Firstly, the findings indicate that manufacturing firms that establish high-quality relationships with approved suppliers' strategic partnerships significantly influence link-coordination and mutual cooperation between the company and its supply chain, ultimately reducing opportunism. Consequently, this study recommends that supply chain practitioners prioritize working with highly trusted, elastic, flexible, dependable, reliable, and engaged suppliers to mitigate supply chain risks associated with raw material sourcing and production requirements. Our results align with many published papers in this topic, suggesting that such relational quality generates value, mitigates and dilutes supply chain risk, and enhances exchange partners' performance [110]. Thus, managers should concentrate on maintaining strategic-term, value-adding, and win-win relationships with dependable suppliers to reap the interests of close and highly relational quality in exchanges. Secondly, enhancing cooperation and diluting supply chain risks can be achieved by improving duple performance between suppliers' strategic partnerships and manufacturing firms and by fostering elasticity and dependability. This involves persistent assist from top-management level and decision makers in implements and activating using quality relationships as a weapon advantage with dependable suppliers and throughout several functions, production processes, and its SC activities particularly with key dependable and reliable suppliers and significant partners. Furthermore, it is suggested that executives in manufacturing firms bolster these collaborative relationships by involving and engaging suppliers, starting from product enhancement and improvement, design phases, making critical decisions for main key partners, and ultimately seeking smart solutions to main problems. This might happen during operational processes to enhance the manufacturing companies’ performance, and their supply chains while diluting potential risks during the supply process.

### 5.2 Research contribution

The manufacturing companies relies mainly on the reliable managing logistical activates through its supply chains to ensure flowing of the required quantities RMs, resources, and items that should be received sites and warehouses at the right place, in right bills, without delay, and with reasonable costs. Therefore, QoMDR, SCRD, and SSP are terms that could support manufacturing companies in improving the effectiveness of their SCPs. SCRD has a direct and significant effect on MFSCP, which is illustrated and approved by research that has been conducted by Ref. [5]. Thus, through formulating a close QoMDR, cooperating, and collaborating with SSP, manufacturing companies will keep ahead of high levels of competition among their competitors, seek new advantages for more success, and confirm leads to enhance their business performance.

This study assesses the effect of adopting QoMDR directly on SCRD, and indirectly through SCRD on MFSCP. Additionally, assesses the direct and indirect impacts of SSP on both SCRD and finally on MFSCP. This paper concentrates on the significance of elasticity, and dependability that is included within the QoMDR construct and SSP of the SCRD of various manufacturing companies in general, and the emerging and developing countries in the Middle East region as reliable sources of supply in particular. Thus, this paper tries to fill some knowledge shortages in the QoMDR domain and its adoption in the emerging and developing countries located in the Middle East region, which can be summarized in the following main points.

➢ After quickly scanning the SCM literature, the fundamental constructs addressed by this paper and concentrated on the SC, which are, QoMDR, SSP, and SCRD in one package research framework and their effects on MFSCP in the emerging and developing countries that are located in the Middle East region, were not covered.
➢ Most previous research papers that were related to this subject partially with some elements have been performed in developed countries, while a limited number of papers have been performed in the Middle East region covering emerging and developing countries. Therefore, this study presents some contributions and fills the gap in the SCM discipline.
➢ A large number of previous studies considered the SSP element to be an effective and important element that plays a role in strategic cooperation if it is optimally employed, and applied with manufacturing companies. Therefore, the findings of this study were identical and reinforced this result, as the SSP factor has a positive and direct effect on reducing potential risks, wasting resources available to companies by activating and applying the SCRD element effectively, which certainly leads to improving MFSCP in general.
➢ There are a significant number of researchers, who did not focus on studying the SCRD component as a mediating variable and the extent of its effect on the relationship with the other factors included in the study between both QoMDR and SCRD on MFSCP. In addition to examining the effect of the SSP element relationship as a moderating variable on SCRD as well.

### 5.3 Limitations and further future research

This paper has some limitations and constraints, which could present some important signals for future work. Firstly, the primary data gathered for this research was sourced from manufacturing firms in only three countries located in the Middle East region (i.e., Jordan, Turkey, King Saudi Arabia, and Egypt), potentially limiting the inclusion of other developing countries. To make our study more international and global, we should consider incorporating additional emerging and developing countries from the Middle East region, and conducting more validation. Secondly, this paper was carried out after an exceptional event that happened in the whole world (i.e., Covid-19 pandemic) in the aforementioned countries (i.e., Jordan, Turkey, King Saudi Arabia, and Egypt), meaning the findings might be not accurately represent the true nature of the associations between the paper main constructs, as these countries continue to face health, social, and economic challenges. Thirdly, this paper did not examine the interactions between local manufacturing firms in these emerging and developing countries and regional or global dependable suppliers, which could be an essential aspect to consider when studying buyer-supplier relationships. Finally, this study relied on data collected from only one respondent, employing a single format approach, which might introduce common bias.

This paper paves the way for academics, practitioners, and scholars to conduct more work and investigations. Considering the outcomes and limitations of this paper, we suggest the following points for future work.

➢ Expanding the scope of this paper by including more developing countries from different regions.
➢ To minimize common method bias, we recommend incorporating a more diverse pool of targeted respondents for data collection and gathering data from various sources, such as suppliers from different countries.
➢ Re-examining the primary dimensions in the proposed model, specifically the relationships included between national, regional, and global providers and dependable suppliers.

## Data availability statement

Data will be made available on request by the authors.

## Funding statement

This study is not funded by any entity.

## CRediT authorship contribution statement

**Moh'd Anwer AL-Shboul:** Writing – review & editing, Writing – original draft, Visualization, Validation, Supervision, Resources, Project administration, Methodology, Investigation, Funding acquisition, Formal analysis, Data curation, Conceptualization.

## Declaration of competing interest

I hereby declare that the disclosed information is correct and that no other situation of real, potential or apparent conflict of interest is known to me. I undertake to inform you of any change in these circumstances, including if an issue arises that is related to my paper.

No funding was received for this work.
